# Cape York Kidney Care: service description and baseline characteristics of a client-centred multidisciplinary specialist kidney health service in remote Australia

**DOI:** 10.1186/s12913-023-09887-6

**Published:** 2023-08-24

**Authors:** Andrea Miller, Leanne Brown, Clara Tamu, Alice Cairns

**Affiliations:** 1 Torres and Cape Hospital and Health Service, PO Box 341, Weipa, QLD Australia; 2 Murtupuni Centre for Rural and Remote Health, & Australian Institute of Tropical Health and Medicine, James Cook University, Cairns, Queensland Australia; 3 Torres and Cape Hospital and Health Service, Ngurapai/Horn Island Primary Health Care Centre, Horn Island, Queensland, Australia

**Keywords:** Chronic disease, Chronic kidney disease, Primary care, First Nations, Indigenous, Multidiscplinary, Rural, Public health, Aboriginal and Torres Strait Islander, Nephrology, Renal

## Abstract

**Background:**

Chronic Kidney disease (CKD) is over-represented amongst First Nation people with more than triple the rate of CKD in those aged 15 years and over. The impact of colonisation, including harmful experiences of health practices and research, has contributed to these health inequities. Cape York Kidney Care (CYKC) has been created as an unique service which provides specialist care that aims to centre the client within a multidisciplinary team that is integrated within the primary care setting of the remote health clinics in six communities in western Cape York, Australia. This research aims to describe the Cape York Kidney Care service delivery model, and baseline service data, including aggregated client health measures.

**Methods:**

The model of care is described in detail. Review of the first 12 months of service provision has been undertaken with client demographic and clinical profile baseline data collected including kidney health measures. Participants are adults (> 18 years if age) with CKD grades 1–5. This data has been de-identified and aggregated.

**Results:**

CYKC reviewed 204 individuals, with 182 not previously been reviewed by specialist kidney health services. Three quarters of clients identified as Aboriginal. The average age was 55 with a high level of comorbidity, with majority having a history of hypertension and Type 2 diabetes (average Hba1c 8.2%). Just under one third had cardiovascular disease. A large proportion of people had either Grade 2 CKD (32%) or Grade 3 CKD (~ 30%), and over half had severely increased albuminuria (A3), with Type 2 diabetes being the predominant presumed cause of CKD. Most clients did not meet evidence-based targets for diabetes, blood pressure or lipids and half were self-reported smokers. The proportion of clients reviewed represents 6.2% of the adult population in the participating First Nation communities.

**Conclusion:**

The CYKC model was able to target those clients at high risk of progression and increase the number of people with chronic kidney disease reviewed by specialist kidney services within community. Baseline data demonstrated a high burden of chronic disease that subsequently will increase risk of CKD progression and cardiovascular disease. People were seen to have more severe disease at younger ages, with a substantial number demonstrating risk factors for rapid progression of kidney disease including poorly controlled Type 2 diabetes and severely increased albuminuria. Further evaluation concerning implementation challenges, consumer and community satisfaction, and health outcomes is required.

## Background

Chronic Kidney Disease (CKD) is associated with significant morbidity and mortality worldwide, with a disproportionately higher impact within vulnerable groups including First Nation’s people [1]. CKD refers to all conditions of the kidneys where there is evidence of impaired or reduced kidney function of at least three months duration [2]. Australian data reveals a higher prevalence of CKD in Aboriginal and Torres Strait Islander people, people from remote communities and socioeconomically disadvantaged populations [1, 3] with CKD in Aboriginal and Torres Strait Islander populations occurring more frequently and with an earlier clinical presentation compared to the general Australian population [1, 4]. Low socio-economic status, remote location, and genomics are key contributing factors to the burden of CKD as well as comorbid conditions of diabetes and cardiovascular disease [1]. Remote Australian communities also have reduced access to specialist care and experience higher mortality [1, 5]. Health care in rural and remote regions is mostly provided through a primary care model whereby general practitioner and/ nurse practitioners provide care within the local communities. Prior to the CYKC service, telehealth was also utilised for review with very high rates of non-attendance. Best practice management of CKD is recommended to be delivered through a culturally safe framework within communities [2].

The western region of Cape York (Western Cape) is a sparsely populated, geographically remote region in far north, eastern Australia. Five of six of the communities in the Western Cape are discrete First Nation’s communities, with a total adult population of 3,296 people [6]. These communities have strong connections to culture, local language and country that are reflected in the community through traditional kinship arrangements, cultural celebrations, and caring for country protocols including fire management, feral species management and pollution control. The strength of the cultural connection contributes positively to health and wellbeing. Despite this, First Nation’s people living in the remote western Cape communities still experience life expectancy twenty years below Queensland average (59 years compared to 80 years), well below national averages [7]. In 2017, a retrospective 12 month audit in the Western Cape, of urine albumin/creatinine ratio and estimated glomerular filtration rate (eGFR) (CKD EPI – ml/min/1.73m^2^) pathology results (n = 1700) indicated a prevalence rate of severely increased albuminuria and/or stage 3 or higher CKD of 2.6% (212 people) out of a total population of 8102 [8]. The incidence of CKD identified by the audit was significantly lower than the estimated 18% with biomedical signs of CKD reported by Australian Institute and Health and Welfare (2023) [9]. These figures indicate that the screening program for CKD within the Western Cape region is inadequate and further efforts need to be made to target CKD and subsequent cardiovascular complications.

Primary care guidelines for the management of CKD recommends referral to kidney specialist services for assessment and treatment planning for people with moderate to severe CKD [2]. Access to kidney specialist services in the Western Cape were delivered in three ways; (1) infrequent, in community face to face reviews required with rotating kidney specialist, (2) travel up to 900 km for face to face review in metropolitan setting, often at great expense to clients and, (3) telehealth reviews facilitated by primary health care clinic. In 2017, only about 26% (n = 56) of eligible clients identified on audit were reviewed by a Kidney specialist service, including Telehealth reviews. This initial review of the size of the health problem and the perceived inadequacy of the existing service model, including lack of community-based services and lack of identification and referral for those with CKD, led to the development of the Cape York Kidney Care (CYKC) model.

Evidence suggests that alongside specialist input, a multidisciplinary team approach has demonstrated a reduction in the progression of CKD, and improvements in all-cause mortality, and mortality due to CKD, compared to a specialist only or general practitioner (GP) led approach [10–13]. Community-based multidisciplinary models are also associated with higher client satisfaction, reduced client waiting and travel times and savings in provider travel costs [12]. Optimal CKD managment programs in community include effective integration with primary care, case management with intensive follow-up, addressing social barriers to adherence, and the provision of culturally safe and appropriate health care [13]. After community and stakeholder consultations, the CYKC service commenced in September, 2019 in the Western Cape, funded by the public health service. This service aims to deliver an evidence-based model by providing care in community. CYKC aims to be holistic and culturally safe, with the intent to improve client engagement, client empowerment and self-management of health,with an emphasis on family and community,. These principles have been demonstrated to be effective in chronic disease models of care [14–17]. Care in the community is a particularly important philosophy of the model, as this allows the local Aboriginal and Torres Strait Islander people to maintain their country, cultural and social/kinship connections [18, 19].

The aim of this paper is to describe in detail the: (a) Cape York Kidney Care service delivery model; (b) baseline client characteristics for the first 12 months of service delivery.

### Situating the research

Although the data presented in this paper is from routinely collected health services records, the data is information about Aboriginal and Torres Strait Islander people who do not have individual agency in how this de-identified information is used. The impact of colonisation, including harmful experiences of health practices and research, has contributed to the health inequities that will be discussed in this paper and the authors are very sensitive to this paradigm. We recognise concerns that Aboriginal and Torres Strait Islander Australians have not seen corresponding improvements in health, despite what many consider is over-researched [20]. The purpose of this paper and publishing this information is not to continue a deficit discourse of the health and wellbeing of Aboriginal and Torres Strait Islander people. Through this focus, this research and described model of care aims to align with decolonising health care practices that recognises strengths, capacity and resilience of communities. Results from this research will be presented back to all individual communities with the focus on reciprocity where the clinical service reported in this paper and subsequent research reflects community priorities and aims to provide a useful service. Outcomes of client and community satisfaction will be formally evaluated in future research and used to inform and adapt this model of care.

## Methods

### Cape York kidney care: clinical model service description

The Cape York Kidney Care (CYKC) model has been designed to be a multidisciplinary service, providing a conduit between specialist-level kidney services and community primary care. The mission statement of the service is: The Cape York Kidney Care team is committed to improving people’s heart and kidney health. We do this by yarning and learning about what is important to you and your family. Together we can empower you to be healthy and live a long life, help to stop the need for dialysis, keeping you connected and communities strong.

The aim of the program is to provide holistic and culturally safe specialist kidney care in community for people identified at high risk of, or with chronic kidney disease. Emphasis is placed on person-centred care and promotion of engagement and self-management of chronic disease via shared goal setting and decision making. The CYKC team consists of a rural general practitioner with advanced skills in adult internal medicine, kidney nurse practitioner, dietitian, Aboriginal and Torres Strait Islander health practitioner and a part-time pharmacist. The service is supported by a kidney specialist based at the regional hospital via teleconference. The combination of a general practitioner with advanced training in adult internal medication, a nurse practitioner with speciality skills in management of CKD and teleconferencing support from a kidney specialist ensures the CYKC model of care provides specialist kidney care within local communities through a primary care model. Team members live and work in one of the Western Cape communities (hub site) and deliver services across the six communities in collaboration with local primary health care centres (PHCC). Although the team has experienced some staff turnover in the first two years, there has always been at least three out of four consistent health care professionals. Five of the six communities have primary care services delivered by the state government as well as an Aboriginal Community Controlled Health Organisation. Two of these communities also have chronic disease services from Royal Flying Doctors – Queensland. Frequency of CYKC clinics range from 1 to 3 monthly. Pharmacist support occurs three to six monthly.

#### Client Population

**Fig. 1:**
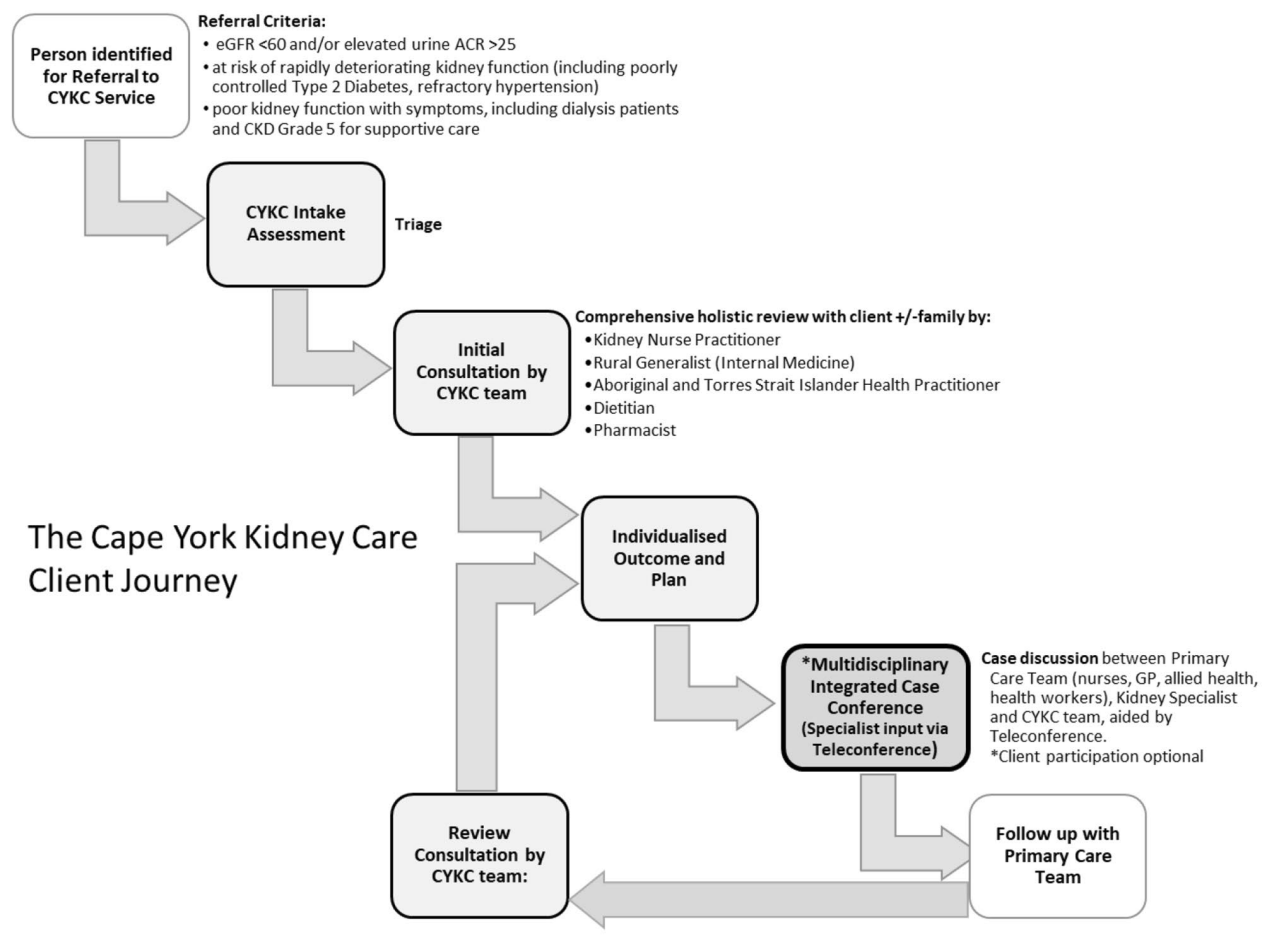
The Cape York Kidney Care client flow

Anyone can access this service if they either are at risk of kidney disease or have evidence of kidney disease as per evidence-based guidelines (see Fig. 1 for referral criteria) [2].

#### Referral process

People either can self-refer or are referred into the CYKC program by any treating health care professional. The CYKC team triage the referral as either category 1 (appointment within 30 days), category 2 (within 90 days) or category 3 (within 365 days) guided by the chronic kidney disease clinical prioritisation criteria utilised in Queensland, however urgency is ultimately decided by the team based on individual factors [21]. See Fig. 1 for the client flow through the service.

#### Service Delivery

Initial outpatient clinic consultation takes place with at least two team members at the clients local PHCC. To reduce the client and family burden of multiple health provider appointments, the clients’ goals and prioritisation of care dictates either sequencing or joint consultations with the most appropriate team members. Elucidating client’s aims, facilitating client engagement and developing a mutual understanding of wellbeing is the priority for initial consultations. If time allows and the client is willing, a biopsychosocial assessment, including symptom review and cultural assessment occurs. The client and their families determine the review interval with team member input. Prior to each clinic, the team liaises with community organisations to engage their support to enable individuals to attend the clinic. Following each clinic, a Multidisciplinary Case Conference is conducted that involves the CYKC team, members of the primary health care centre (including nursing staff, allied health, general practitioners, and Aboriginal and Torres Stait Islander health workers), and the kidney specialist, facilitated by teleconference (Fig. 1). A management plan is generated at the conclusion of this meeting and circulated to all members of the wider health care team, with actions assigned accordingly, and a copy offered to the client/family member. Telehealth is also occasionally used as an adjunct in the case that a CYKC team member is not able to review a client face to face, but could not be relied on as sole method for client review (i.e. at least one team member would be present with the client). The CYKC team also offers inpatient reviews of shared clients at the ‘hub site’ hospital and ongoing ad-hoc outreach support to the six clinics and direct to individual clients.

### Study design

This is a cross-sectional investigation of consecutive clinical charts from initial CYKC consultations.

### Procedure

Initial intake, assessment and administrative service data from consecutive charts were audited by one member of the research team with support from a second member to check and clarify any ambiguous data. Basic demographic and clinical data including; age (in years), sex *(M/F/UK)*, ethnicity (self-reported), diagnosis of Type 2 Diabetes Mellitus (T2DM) *(Y/N)*, presence of cardiovascular disease *(Y/N)*, use of antihypertensive therapy *(Y/N)*, use of oral hypoglcaemic therapy *(Y/N)*, grade of CKD as defined by estimated glomerular filtration rate (eGFR) mL/min/1.73m2 [22], presence of severe range albuminuria > = 30 mg/mmol [22], presence of hypertension *(Y/N)*, defined as blood pressure over130/80 [2], glycated haemoglobin (HbA1C) (mmol/mol), hypercholesterolaemia *(Y/N)* defined as total cholesterol over 5 mmol/L [22], and smoking status *(Y/N)*. All data has been aggregated and descriptive statistics used to report results.

## Results

### Client demographics

Over the first 12 months of the CYKC service, 204 people engaged with the service. The average age of clients was 56 years old (range 18–93 years), with a majority (92%) of clients identifying as Aboriginal and/or Torres Strait Islander. Almost half of all clients (48%) reported to smoke cigarettes. Demographic and clinical characteristics of the client group are presented in Table 1.

**Table 1:** Demographic and clinical characteristics of clients in the first twelve months of service n = 204

Demographics	N (%)
*Sex*	
Male	94 (46)
Female	110 (54)
*Age*	
18–34	14 (7.3)
35–49	36 (17.7)
50–64	97 (47.5)
> 65	56 (27.5)
*Ethnicity*	
Aboriginal	153 (75)
Torres Strait Islander	14 (6.9)
Aboriginal and Torres Strait Islander	19 (9.3)
Non-Indigenous	8 (3.9)
Non-Indigenous (Other ethnic minority)	8 (3.9)
Unknown	2 (1)
**Comorbidities**	**N (%)**
Type 2 Diabetes	151 (74)
Hypertension	185 (91)
Cardiovascular disease	55 (27)
Hypercholesterolaemia	159 (78)
**Clinical characteristics**	**Mean (range)**
uACR (mg/mmol)†	140.2 (0.3–1300)
eGFR (mL/min/1.73m2)	61 (5 - >90)
Hba1c (%)§ Hba1c (mmol/mol)	8.6 (5–15.2) 70 (30 - >108)
Systolic Blood Pressure (mmHg)	139 (90–223)

†Urine Albumin-to-Creatinine Ratio uACR (n = 162), § Diabetic cohort only (n = 151); Hypertension (n = 177 previously diagnosed) and (n = 8 new diagnoses) with systolic blood pressure > 130 at baseline; Hypercholesterolaemia (n = 125 previously diagnosed) and (n = 34 new diagnoses) with total cholesterol over 5 mmol/L.

### Clinical characteristics

As displayed in Table 1, the majority (n = 185) of clients had hypertension (either newly diagnosed at time of review or previously identified). Of the 177 clients with previously identified hypertension 59 (33%) had achieved a blood pressure less than 130/80 at time of review. Type 2 Diabetes was prevalent at a rate of 74% (Table 1), with 29% of diabetics treated to Hba1c < 7.0%. In the diabetic cohort, the average Hba1c was 8.6% with a range of 5.0 to 15.2%. A history of cardiovascular disease was identified in 27% of clients, with 23% having both cardiovascular disease and Type 2 diabetes. Cholesterol of greater than 5.0 mmol/L was identified in 37% of clients at time of initial review, with 60% of these being recommended for statin therapy.

**Fig. 2:**
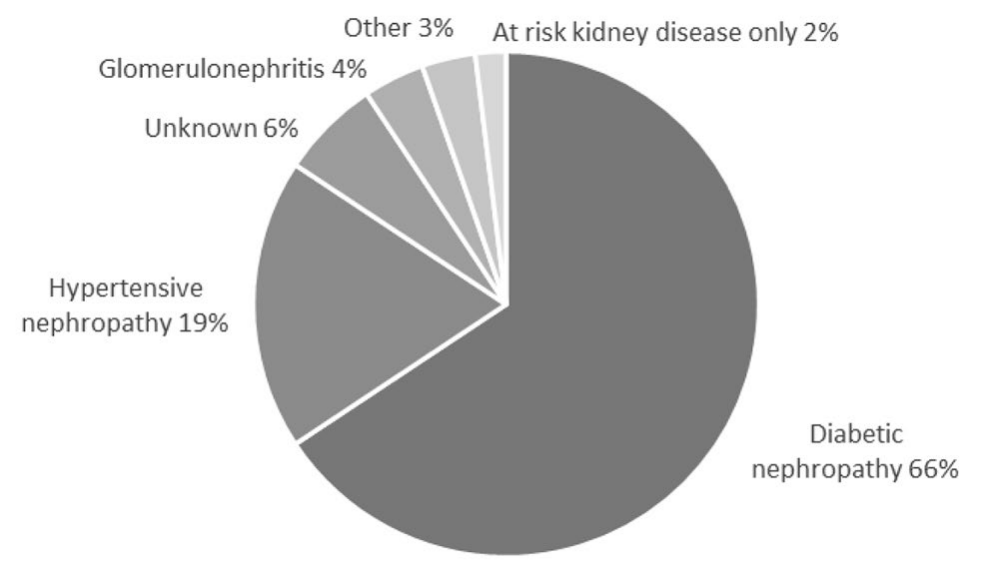
Presumed aetiology of chronic kidney disease by percentage (n = 204)

**Fig. 3:**
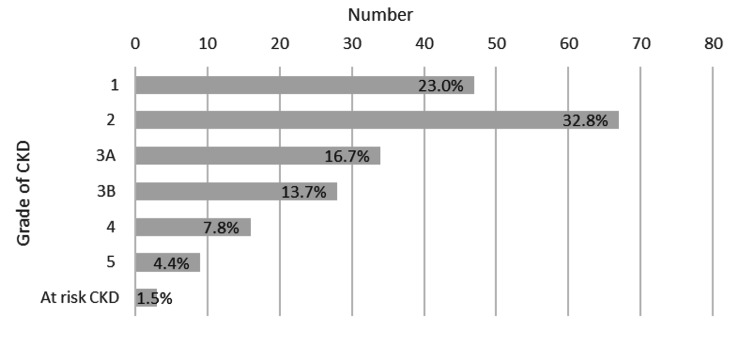
Number and percentage of total people accessing CYKC service by grade of CKD (n = 204). Grade 1 eGFR > 90ml/min/1.73 m, Grade 2 eGFR 60–89, Grade 3 eGFR 30–59, Grade 4 eGFR 15–29, Grade 5 eGFR < 15 [22]

**Fig. 4:**
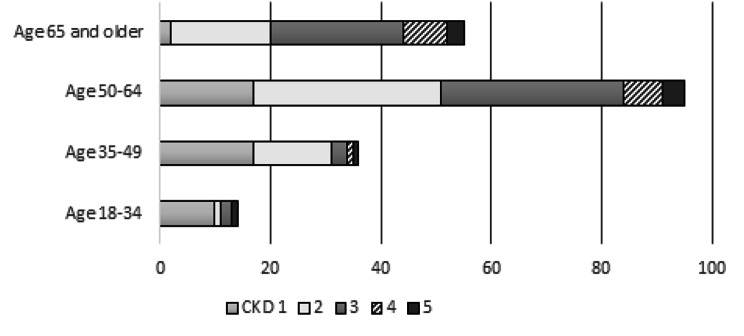
Distribution of age and CKD Grade for people accessing CYKC service (n = 204)

The predominate primary presumed diagnosis was diabetic nephropathy (66%) followed by renovascular disease (hypertensive nephropathy) (19%) (Fig. 2). The distribution of the grades of CKD is presented in Fig. 3, with a majority of the population identified as grade 2. The average eGFR was 61 ml/min/1.73m^2^. The age groups of 18–34 and 29–34 years had higher proportion of early CKD with higher distribution within Grade 1 CKD (Fig. 4).

**Fig. 5:**
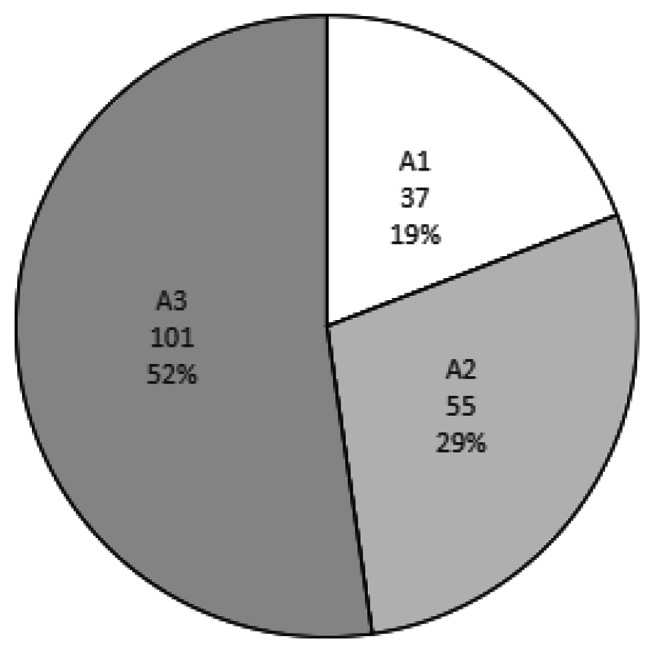
Baseline albuminuria range for people accessing CYKC Service (n = 193). A1 No albuminuria (uACR < 3.0 mg/mmol); A2 Moderately increased albuminuria (uACR > 3.0–29 mg/mmol); A3 Severely increased albuminuria (uACR > 30 mg/mmol)

There was a large proportion (51%) of people with severely increased albuminuria (> 30 mg/mmol) (Fig. 5), with 48.5% of these people in early stages (Grade 1 and 2) of CKD Of the total cohort, 17.1% had nephrotic range uACR > 299 mg/mmol [22].

## Discussion

The primary purpose of the CYKC service is to provide access to specialist care in the clients’ local community. In the first 12 months, 204 clients were reviewed by CYKC, initially high-risk clients were invited to the service based on pathology audits, however as primary care services were socialised to the model, referrals accelerated beyond expectations. As this is a new service, it is recognised we have yet to capture the full population that are eligible for the service however compared to previous service delivery data from 2017 the number of people accessing kidney specialist service has quadrupled, 56 people in 2017 to 204 in this 12 month period. The number of clients reviewed within the first twelve months represents 6.2% of the adult population in the participating First Nations communities. As indicated by AIHW the estimated proportion of First Nation peoples with CKD is approximately 18% [9]. Thereby identifying that the CYKC service at 12 months has captured one third of those with CKD. Future research will explore longitudinal service utilisation and client outcomes.

The referral criteria for this service aims to target early CKD and risk factors of CKD which represents a different approach to Kidney Disease Improving Global Outcomes guidelines referral criteria [22]. The key difference is inclusion of clients with elevated HbA1C in absence of signs of CKD or with only mildly increased albuminuria. The intent is to provide preventative interventions earlier to reduce the rates of CKD, which can lead to rapid progression in the Aboriginal and Torres Strait Islander population [23]. Early identification and management typically happen at the primary care level, [24] in northern Australia this involves GP’s, rural nurses and Aboriginal and/or Torres Strait Islander health workers. The results from this study have identified reasonable screening and identification of clients with hypertension, hyperlipidaemia and diabetes (risk factors for CKD) at the primary care level, however there was inadequate management, with only a third reaching the recommended blood pressure target of 130/80, and the average Hba1c considerably higher than evidence-based targets. Hyperlipidaemia represents a further risk factor for the CYKC population with baseline data identifying less than half of the cohort being treated to target total cholesterol, and a subset not on appropriate pharmaceutical treatment when considering presence of moderate to severe proteinuria.

The prevalence of co-morbidities at baseline is similar to other kidney health studies, including the CKD.QLD dataset [25]. Similarly, a large study undertaken in general Asian populations found comparable overall incidence of hypertension [26]. The rates of type 2 diabetes (74%) was unsurprising but of concern given the high rates (69%) of diabetic nephropathy among the dialysis population (69%) [27]. Other population-based kidney health studies identified similar rate of hyperlipidaemia but the association between moderate albuminuria and prescription of statin therapy has not been examined [28].

Early identification of CKD, risk stratification and implementation of interventions is critical in the management of CKD however the service model to deliver this management has not been adequately determined nor evaluated [29]. Examination of the clinical characteristics in this study helps to identify current primary care management of CKD and associated risk factors in the participating communities. The participant group in this study represents about 6% of the total adult population of the First Nations communities in the Western Cape. Within the participant group, the incidence of moderate to severely increased albuminuria was nearly half of the population with a proportion (17.1%) exhibiting nephrotic range proteinuria. Other recent CKD population-based studies found a similar incidence of moderate to severe albuminuria [25, 30]. These results are in contrast to an earlier population-based study undertaken in the general population of Australia where only 0.7% of the population had moderate to severely increased albuminuria [31]. The high incidence of severely increased albuminuria represents a high risk of cardiovascular mortality and kidney disease progression [22]. Key management strategies to reduce albuminuria involve management of blood pressure, Type 2 Diabetes, hyperlipidaemia and include targeted lifestyle interventions [22], as well as evidence-based use of medications such as angiotensin-converting enzyme or ACE inhibitors, aldosterone antagonists and more recently sodium-glucose co-transporter 2 inhibitors [2]. Health services in Australia have implemented screening programs and surveillance programs [32] without adequate evaluation of CKD models of care to determine effectiveness.

In First Nation’s communities’ significant barriers are presented due to the lack of culturally safe and appropriate health care, and failure to meet client-prioritised health outcomes [19]. The CYKC model has attempted to overcome some of these barriers by focusing on tools that have been shown to improve primary care delivery including supportive technology and collaborative relationship between members of the healthcare team [33]. In particular, the role of the Aboriginal and Torres Strait Islander health practitioner is important in ensuring cultural safety of clients accessing the service, allowing for cultural brokerage and client advocacy [34], and providing education about kidney health in a culturally and language-appropriate manner, promoting client understanding of health and improving agency and self-empowerment. The model also works to promote best-practice CKD management by educating the primary health care teams through vertical and horizontal translation of knowledge [35, 36]. Understanding baseline characteristics of CYKC population will help to identify the gaps in current service for people with CKD.

With respect to lifestyle measures, approximately half of the cohort were smokers, consistent with other data where rates of smoking amongst Indigenous people is significantly higher than non-indigenous counterparts (5% nationally) [37]. Data for other lifestyle measures such as nutrition, alcohol consumption and physical activity was not consistently collected and has not been reported on and data on body mass index was not collected reliably. This is a weakness of this baseline data and difficulties with collecting this information is due to often ad hoc clinic environments and lack of access to necessary equipment, a reflection of difficulty of health service logistics in remote settings.

The CYKC program has many elements that have been designed to contribute to the model’s success, including integration within existing services, a team approach, intensive follow-up, and a prioritisation on clinical systems supporting communication [38].The consistency of staffing is a considerable strength of the service model as it has afforded the time required to build trust and relationships in the communities with clients and family members, and primary care staff. The presence of clinical staff (e.g. nurse practitioner, GP with advanced internal medicine) working to advanced scope of practice within the CYKC model [39–41], longer consultation time [40], comprehensive clinical notes and shared management care plan are key features of successful chronic disease management models [40]. In addition, it has also been demonstrated that provision of culturally appropriate education and a central role for Aboriginal and Torres Strait Islander Health Workers are important for ensuring engagement and improving access [38]. The limitation to the CYKC model is the dependency on small number of staff for service delivery in outreach communities. Consideration must occur as how translation of knowledge to the primary care members can reduce the need to CYKC teams members to review clients as frequently.

Despite all the potential strengths and intentions of this model, it is a medical solution to predominately a social problem that operates in a neoliberal environment. The individual, who experiences considerable social discrimination through historical and ongoing colonialisation, becomes responsible for making changes to improve medical markers of wellbeing. Well-meaning funding of health services that are striving for more integrated and culturally safe delivery are admirable but remain a very costly way to provide access to care which could have been avoided. Research priorities for improving health outcomes through improvements in the social determinates are yet to be translated into action [20]. This baseline review of data provides evidence for urgent action from national, state and local entities to prioritise and collaborate on structures to improve the social determinants of health.

## Conclusion

A community-focused model has been developed to improve access to specialist kidney services to very remote Australian First Nation’s communities, however not yet fully capturing predicted population with biomedical signs of CKD. Baseline data demonstrates that there is a significant and disproportionate burden of chronic kidney disease and its co-morbidities (Type 2 diabetes, hypertension and cardiovascular disease) at a younger age in people living in remote Far North Queensland, Australia and that chronic disease continues to be inadequately managed at the primary care level. Strengths of the model include its ability to integrate into current primary and tertiary systems of care. Improving social determinants of health, including access to adequate nutrition and housing, and addressing disproportionately high smoking rates are critical in improving health outcomes in remote First Nation’s communities. Further evaluation will be undertaken via qualitative and quantitative research methodologies. This research will consider implementation challenges, consumer and community satisfaction, and health outcomes.

## Data Availability

The data that support the findings of this study are available from Torres and Cape Hospital and Health Service. Restrictions apply to the availability of this data, which were used with permission for the current study, and are not publicly available. Data are however available from Dr Andrea Miller (author) upon reasonable request and with permission of Far North Queensland Human Research Ethics Committee and Torres and Cape Hospital and Health Service.

## Abbreviations

CKD: Chronic Kidney Disease
CYKC: Cape York Kidney Care
Hba1c: Glycosolated Haemoglobin
eGFR: Estimated glomerular filtration rate
mL/min: Millilitre/minute
GP: General Practitioner
PHCC: Primary Health Care Centres
M/F/UnK: Male, Female or unknown
Y/N: Yes/No
T2DM: Type 2 Diabetes Mellitus
uACR: Urine Albumin-to-Creatinine Ratio
